# Radioactivity to Rethink the Earth's Energy Balance

**DOI:** 10.1002/gch2.202000094

**Published:** 2021-03-17

**Authors:** Maria Letizia Terranova

**Affiliations:** ^1^ Tor Vergata University of Roma Department of Chemical Sciences and Technologies Via della Ricerca Scientifica Roma 00133 Italy

**Keywords:** eumelanin, nuclear batteries, nuclear waste, power plants, radioactivity

## Abstract

This contribution invites to re‐examine the whole matter of radioactivity, reconsidering it from the point of view of a realistic source of energy. State‐of‐the‐art and technical aspects are briefly illustrated in this note that aims to open a discussion on this challenging topic.

## Graphical abstract

This comment aims to re‐examine the whole matter of radioactivity, from the perspective of the current need to recycle for a sustainable development. In this frame, natural radioactive sites and storage areas of nuclear waste are sources of “endless” available energy to be safely re‐used. State‐of‐the‐art, technical aspects, and obstacles stemming from social perceptions are briefly illustrated.

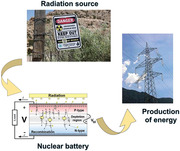

The first alarm about the criticalities that could arise out of an uncontrolled development of technology has been given by the Club of Rome, that in 1972 published the famous report “The Limits to Growth”.^[^ ^1^ ^]^ This fundamental study started to question the paradigm that the humanity had undefined available resources and could therefore perturb the ecological equilibrium without consequences.

The industrial era has been indeed characterized by an antagonism between environment and technology, resulting in the production of artificial objects and anthropomorphic systems with no analogs in nature, and in the disruption of any balance between resources and energy consumptions. We are now well aware of the fact that the limited supply of the fossil energy sources cannot meet future needs. Moreover, fossil fuels are the major responsible of the abnormal climate changes, closely correlated to a CO_2_ emission unparalleled over the past. It is now recognized that the right approach to manage our world, responding to the sustainability challenges of the XXI century, is the development of strategies to restore a virtuous cycle of energy/matter inter‐change. This consciousness is leading today to a radical departure from conventional solutions toward innovative processes, attempting to model man‐made systems on nature‐like ones.

Millions of year of evolution allowed nature to find the best solutions for a series of problems, such as the energy requirements of the biosphere. For the vegetable kingdom the solution was the photosynthesis process, that converts the solar energy into metabolic energy.

## 1 Background

In the search of renewable innovative energy sources, scientists tried to replicate the photosynthesis process by assembling photovoltaic cells based on semiconductors. In particular the dye‐sensitized solar cells proposed by Gratzel in 1991^[^ ^2^ ^]^ mimic the methods adopted by nature that uses complex bio‐organic structures for light absorption and energy production. In the “Gratzel‐like “devices the role of chlorophyll to transform photons into chemical energy is played by artificial dyes that, coupled with semiconducting materials, generate electrical power.

However the opportunity to convert sunlight into available energy, either metabolic or electrical, is offered only to systems exposed to the sun radiation, whereas we know that there is life also in regions isolated from the photo‐sphere. Under such conditions life evolved developing alternative mechanisms and harvesting energy from different natural resources.^[^ ^3^ ^]^ As disclosed by paleo‐biological investigations, the exploiting of natural radioactivity was one of the other way followed from million years by biosystems for life flourishing in absence of solar light.^[^ ^4^, ^5^ ^]^

When radioactivity comes into play, a generalized anxiety is generated, because people connect such physical phenomenon to the big catastrophes that occurred in the recent years (Chernobyl, Fukuyama). But the radioactive decays are physical processes naturally occurring “on” and “around “the earth. Mankind coexists ab initio with radiations emitted from terrestrian rocks and landed meteorites, as well as with cosmic rays raining down on the Earth.^[^ ^6^ ^]^ On the other hand the advent of the nuclear era and the related escalation of civil and military applications led to a continuous production of artificial radioisotopes, either obtained as by‐products of fission reactions or purposely generated for specific applications, as diagnostics and medical therapies.^[^ ^7^ ^]^ In each case the safe disposal of such radioisotopes, that can remain radioactive up to tens of thousands years, is a problematic task and the produced nuclear waste is an additive source of environmental radioactivity. From the point of view of safety the nuclear decays cause endless troubles but, from a different perspective, radioactivity can be viewed as a resource in the global Earth's energy balance.

The idea to utilize highly energetic emissions from radionuclides is not new. The concept of a nuclear battery was first proposed by H.G.W. Moseley, who in 1913 fabricated and tested a device exploiting the energy released in the α‐decay of natural Radium.^[^ ^8^ ^]^ Further research work demonstrated that nuclear batteries could generate electricity by converting highly energetic alpha (α) and beta (β) particles as well as gamma (γ) radiations emitted from a variety of radioactive isotopes.

A complete analysis of the possible radiation sources to be utilized for energy harvesting can be found in ref. ^[^ ^9^ ^]^. The article by Prelas et al. takes into consideration, besides the α, β, and γ nuclear decays, also the emissions of neutrons and fission fragments, and illustrates the feasibility to fabricate nuclear batteries able to produce electricity by a series of different processes. As described in ref. ^[^ ^9^ ^]^, the generation of electricity from radioactivity can indeed be obtained by means of various thermal and non‐thermal conversion mechanisms, leading to a variety of devices suitable for different applications. The success demonstrated in numerous deep space missions by the isotopic thermoelectric systems, that generate power using the heat produced by radioisotope decays, has set a performance standard that serve as reference for all kind of nuclear batteries.^[^ ^10^ ^]^

A non‐thermal solid‐state device that produced electricity from the β‐induced ionization of intrinsic semiconductors was patented in 1953.^[^ ^11^ ^]^ In the β‐voltaic battery a direct energy conversion was achieved using the converter in a conventional p‐n diode configuration, where electron–hole pairs are generated and electrons are swept across the electric field. From the ‘70s to late ‘80s, β‐voltaic cells based on the use of long lived radionuclides (mainly 147‐Pm and 238‐Pu) have been widely used to power pacemakers.^[^ ^12^ ^]^ In the implanted patients such devices showed an extraordinary reliability combined with proper functionality and lack of safety risks. The current replacement of the nuclear cardiac devices with Li‐powdered batteries has being motivated by other concerns, related more to security than to safety.^[^ ^13^ ^]^

Conversely, from the beginning the technology of batteries based on α‐particles emitted from radioactive nuclides faced some difficulties.^[^ ^14^ ^]^ In effect, wide band‐gap and radiation‐tolerant semiconductors are needed to convert the α‐decay into electrical current, but the energy output of an α‐voltaic device is a factor ≈100 greater than that of a similar β‐voltaic power source (assuming the same conversion efficiency).

Overall, all the nuclear batteries based on the direct conversion mechanism offer energy densities higher than any other power source,^[^ ^15^, ^16^ ^]^ as shown in the plot of specific energy density (J kg^−1^) against specific power density (W kg^−1^) reported in reference ^[^ ^16^ ^]^.

However, the nuclear batteries with long shelf‐life suffer from some intrinsic limitations, such as low specific power density, low efficiency (typically <10%), and radiation damaging of the converter material.^[^ ^9^, ^17^ ^]^ Moreover there are obstacles in cell size decreasing.^[^ ^9^ ^]^ The effective miniaturization of nuclear devices is nowadays a virtually unattainable objective, mostly when the highly penetrating γ ‐rays are the radioactive source and a proper shielding is needed. The failing to down‐scale batteries size is a critical constraint that virtually hampers the integration in the ever‐smaller and extremely compact electronic devices used in several technological fields. The whole of shortcomings is nowadays restricting the usage of nuclear batteries only to some niche applications.^[^ ^18^ ^]^ In particular such energy sources are employed in space missions or in sensors and communication devices/nodes located in remote or harsh environments, which are required to last the lifetime of the infrastructures.^[^ ^19^, ^20^ ^]^

Nevertheless, the advantages offered by the long lifetime is still pushing researchers to improve the performance of radiation‐based solid‐state systems, making them appropriate for a wider range of end‐uses. Recent studies demonstrated that the choice of a proper semiconducting material, together with a non‐conventional design of the converter, can enhance the electrical power output. The specific energy of a β‐battery based on diamond in a Schottky diode configuration can indeed reach values an order of magnitude higher than those of a conventional chemical cell.^[^ ^21^ ^]^ Up to now several converter configurations have been tested, producing devices with innovative quasi‐vertical, vertical, and also “corner” architectures.

Even if there are no doubts that optimization of the cell design and new fabrication routes will in future improve the performances of such batteries, the combination of downsizing constraints, low conversion efficiency, and limited power density is hindering a large scale commercialization of nuclear batteries for integration in tiny electronic devices. But the above mentioned drawbacks are really limiting the possibility to use nuclear radiations as an alternative energy source?

## 2 Chances

Thinking to the abandoned mines of U, Th, and rare earths elements (REE), or to the storage areas of spent nuclear fuel and artificially produced radioisotopes, it is clear that such premises could be regarded as rather “endless” sources of an energy that wait only to be properly captured. When available and affordable sources of radiation are put into play and a unexhausted supply of high‐energy radiations is guaranteed, all the drawbacks experienced in harvesting power by means of nuclear batteries are expected to fade.

One could envisage indeed the fabrication of centralized utility‐scale installations where a large number of converters can be connected in modules, overcoming the low efficiency of the direct or un‐direct mechanisms of conversion. In such plants, fabricated inside abandoned mines of U, Th, and REE, or near storage areas of artificially produced isotopes, the battery miniaturization would be no more a concern. By the way, one would no longer speak of true “batteries”, that is, of the complete systems that supply electricity by converting energy from an internal radioactive source, but merely of “converters”, that is, the semiconducting component that acts as power generator.

As regards the radioactive sites, there is certainly an ever growing public concern about them, but one can note that not all the radiation sources are looked at with the same attention. Storage sites of radioactive waste produced in nuclear plants face effective protest by citizens and the risk perception influences negatively the public acceptance, even if safety in these installations is assured by severe regulatory issues. Conversely, little or at all no attention is paid to the mining sites of radioactive minerals and to the related issues of safety, environmental effects and also security. In this context one must figure out not only the operating mines, but rather the disused ones. Around the world there are indeed a lot of old U, Th, and REE mining areas that, after the closing of exploitation, have been abandoned by the companies without any on‐ site recovery. The abandoned premises and their surroundings represent a strong hazard for the local communities. As in the case reported in,^[^ ^22^ ^]^ often the populations are exposed to radiation doses that exceed the reference values for annual effective limit.^[^ ^23^ ^]^

The mitigation of the effects produced on both population and environment would need a deep rehabilitation of the legacy mines and a complete containment of the radioactive areas. However such topic does not affect the public perception, therefore the protection against radiations in former mines is not seen as a pressing objective by the opinion‐makers of the environmental movements. In this view, the putting in place of costly recovery strategies is very unlikely, unless outstanding economic interests were coming into play. The planning of centralized battery banks managed through validated protocols would provide the positive side‐effect to assure public health, environmental safety, and security.

As regards the transducers, the obstacles found when using conventional semiconductors can be overcome by experimenting alternative materials. In this context a brilliant example is given by the use of radiation‐tolerant artificial diamonds for the assembling of nuclear‐powdered batteries prototypes.^[^ ^21^ ^]^

However, also other materials can offer solutions for fabrication of durable transducers to be interfaced with radiation sources. Surprisingly, some of the more radiation resistant semiconductor materials are biopolymer found in living species.

In recent years it has been discovered that some organisms growing in radioactive environments utilize radioactivity as a source of metabolic energy. Such unexpected behavior was preliminary noted at the end of 50's in fungal colonies grown in Nevada nuclear test sites.^[^ ^24^ ^]^ More recently it was discovered a flourishing of single‐cell fungi in the highly radioactive areas surrounding the damaged Chernobyl Atomic Energy Station or in the cooling water of still operating nuclear reactors.^[^ ^25^ ^]^

The studies on the species dominant in soils contaminated by naturally occurring or anthropogenically originating radionuclides, as well as in the natural high‐radiation environment (i.e., Antarctica highlands) enabled to disclose that in all cases such species were rich in melanins.^[^ ^24^, ^25^ ^]^ The broad term “melanins “indicates a class of naturally occurring conjugated polymers, based on C_18_H_10_N_2_O_4_ molecular sub‐ units, widely present in living organisms.^[^ ^26^ ^]^

Whereas at the beginning of 2000's it was well known that eumelanin, the most interesting component of the melanin family, has the capability to absorb a wide range of the electromagnetic spectrum, at that time there were only hypothesis about the mechanism followed by this biopolymer to transform dangerous α, β, and γ radiations in energy for physiological processes. Evidence proving the role played by eumelanin in the radiation‐induced increase of metabolic activity was achieved from laboratory studies that highlighted modifications in the irradiated cells of the eumelanin electronic structure.^[^ ^27^ ^]^ Attempts to understand the way radioactivity is transformed by eumelanin in energy available for metabolic processes evidenced similarities with the mechanisms adopted by chlorophyll in turning solar energy into bio‐energy.^[^ ^28^, ^29^ ^]^

In both cases what come into play is the electronic structure of the chemical species, but, whereas chlorophyll generates chemical energy from non‐ionizing radiations through the photo‐synthesis process, eumelanin carries out radio‐synthesis processes, converting in chemical energy both the ionizing high‐energy portion of the electromagnetic spectrum and the radiations from nuclear decays.

The electronic properties and the semiconducting behavior of eumelanins were first outlined in 1972 by J.E. McGinness who tested the material as an amorphous semiconductor threshold switch.^[^ ^30^ ^]^ At that time the conductivity was explained in the frame of the amorphous semiconductor model and of the modified dielectric theory.^[^ ^31^ ^]^ Afterward a number of observations evidenced a hydration‐dependent charge transport mechanism and suggested for eumelanin a role of hybrid ionic–electronic conductor. The conduction behavior of these macromolecules is presently described in terms of an interplay between proton migration, redox processes, and electronic transport.^[^ ^32^ ^]^

The studies on the charge transport and energy transductions allowed biologists to reshape the conventional schemes about electron transfer in metabolic pathways and to advance hypothesis about the mechanisms implemented by eumelanin to assure radiation resistance in living organisms.^[^ ^29^, ^33^ ^]^ It is now confirmed that the paramagnetic eumelanins succeed in quenching the cytotoxic free radicals produced by radioactivity by putting into action their complex free radical system.^[^ ^34^, ^35^, ^36^ ^]^

The hybrid electrical conduction coupled with an efficient free‐radical scavenging mechanism^[^ ^35^ ^]^ makes eumelanin capable to parallel, or also to go beyond, the performances of inorganic amorphous semiconductors. In this view the viability of an eumelanin‐based bio‐electronics and ‐optoelectronics has been largely investigated,^[^ ^37^, ^38^, ^39^ ^]^ and guidelines to improve charge transport features of such organic semiconductors are now provided.^[^ ^40^ ^]^ Nevertheless, whereas a lot of significant applications have been proposed for bio‐electronics and biosensing,^[^ ^41^, ^42^, ^43^ ^]^ the issue of energy harvesting by eumelanin‐based devices is rather overlooked by the scientific community. This is likely due to the output of some studies that evidenced an intrinsic low conductivity of the material, poor performances in converting light into electricity, and a scarce stability of eumelanins under long‐term illumination.^[^ ^44^ ^]^

However such drawbacks regard the efficiency of light conversion, in which eumelanin is certainly not competitive with inorganic and also some organic semiconductors. If ionizing radiations are taken into account as energy source, the approach must be completely reversed. It is indeed well known that ionizing radiations have a strong detrimental impact on inorganic semiconductors and the low resistance to radiations of conventional semiconductors is precisely one of the arguments that may hinder the development of nuclear batteries.^[^ ^9^, ^45^ ^]^ On the contrary, eumelanins could be a successful active semiconducting material in converter devices able to generate power also from highly radioactive sources.

In the last decade the traditional picture of eumelanin as the pigment absorbing harmful components of the solar spectrum has been widely broadened, to include the unexpected features of radioprotection. The scouring of the biomedical literature allows one to find many studies and commentaries that discuss the role of melanin in mitigating the effects exerted by radiation, radioisotopes, and fallout on living species. Eumelanin‐rich fungi have been tested as radioprotective food^[^ ^46^, ^47^ ^]^ and ESA recently funded a project to evaluate eumelanin‐based materials for the human protection during space flight missions.^[^ ^48^ ^]^

What are missing, conversely, are studies/speculations about the possibility to capitalize on the opportunities that eumelanin offers for electrical power production from radioactive sources. It is to be noted that from a technological point of view there are no stumbling blocks hampering the realization of safe and sustainable energy sources from nuclear decays. Moreover the current state‐of‐art can foster the tackling of such issue in a reasonable development timeline. Some U.S. companies, like City Lab and Widetronix, have been developing commercial β‐voltaic cells for decades. Nowadays, the task to optimize both battery design and converter materials is being addressed in several research centers, where alternative solutions to the experienced drawbacks are actively explored. In the search of the best solution, the researches encompass a variety of materials with different structures, mechanical properties, and energy gap. Besides the already cited diamond, also eumelanin is being taken into account as a material suitable for radioactive conversion.

## 3 Outlooks

Overall, there are no doubts that the advances in materials science and technology will allow to identify and produce materials and systems suitable also for the scaling‐up of the processes. Other are the issues that can really impede to attain the objective to produce a “carbon‐free” energy, converting in a resource the trouble connected with already existing radioactive sites. The first one is a somewhat compartmentalization of the researches. It is to be noted that each topic briefly outlined in this paper, namely radioactivity, nuclear batteries, and eumelanins, has been—and still is—the object of deep investigations carried out in the frame of highly specialized and sectorial scientific/technological areas.

As an example, the idea of energy harvesting from radioactivity, launched inside the restricted community of researchers working in nuclear technologies, did not trigger up to now the attention of people addressing the topic to rebalance energetic cycles and processes for a sustainable development. The management of the fast changes of today's world and the tackling of society's grand challenges must pass through the strategic priority to create a fertile ground for really multidisciplinary collaborations covering the whole spectrum of technologies.

The second big issue is the negative perception and subsequent low acceptance of radioactivity, even of the natural one, by the general public. It is to be noted that the concern about radioactivity in our society is greater than the actual health hazard. This depends mainly on the lack of a correct scientific information necessary to overcome the generalized fear created by the catastrophic events of the last decades. By the way, general public not only ignore the annual collective radiological dose produced by exposure to radiations from environment (cosmic rays, ground, and food/waters) but also the fact that devices and systems based on radionuclides are largely present in almost all technologies, even those considered eco‐friendly or green. Various non‐nuclear energy industries rely indeed on REE and radionuclides to produce primary forms of energy, to increase the energy efficiency or to assure long life‐times for security systems (examples: Radio‐luminescent devices, smoke detectors).^[^ ^49^ ^]^ The matter to overcome irrational concerns and to earn the public's trust should be addressed by launching initiatives and promoting effective information campaigns about radioactivity and risk assessment.

Another positive step in this regard could be taken by evidencing the salient socioeconomic benefits provided by the re‐use of radioactive sources. Such a topic could be identified and presented as an implementation of the nowadays generally accepted philosophy of sustainable development. The harvesting of energy from already existing radioactivity is to be seen indeed in the context of the “re‐use and recycle” imperative need of our society. Looking at the nuclear waste in the frame of the leading theme of a global waste management would significantly help in changing the public attitude. Remarks concerning the recycling value chains must be formulated in such a way to persuade people that the challenge to increase the worldwide energy yield with benefits for environment, economy, and territories needs a strong commitment planning and the development of even unconventional technical solutions.

The overcoming of conceptual barriers in the public opinion would also result in a better acceptance of other nuclear‐related innovative energy systems, as the distributed nuclear power reactors.^[^ ^50^ ^]^ Nowadays, the advances in nuclear technology are indeed allowing the design of microreactors and also of mobile reactors able to overcome many of the constraints and drawbacks of conventional utility‐scale nuclear plants.^[^ ^51^ ^]^

The projections indicating attractive future markets for atomic batteries^[^ ^45^ ^]^ and the strong interest of many energy‐supplying industries for the new concept of distributed nuclear reactors^[^ ^50^ ^]^ give hope that someday the public opinion will no longer represent an insurmountable obstacle in the use of nuclear sources to address a sustainable electrification.

At this stage of global systems development, when “what to do” for decarbonization is a challenge of the outmost importance, and current technologies are ready for nuclear‐based innovative solutions, the issues of non‐segmental knowledge and of a new social consciousness are strategic priorities. As indicated in the more recent report of the Club of Rome^[^ ^52^ ^]^ collective actions must be planned by governments through the cooperation of experts on technologies, policymakers, climate advocates, and consumers. To conclude, a convergence of technologies and social sciences is needed to realize that, in order to satisfy the requirements of our energy‐consuming world, we must not disregard any available source of energy.

## Conflict of Interest

The author declares no conflict of interest.

## Data Availability

Data sharing is not applicable to this article as no new data were created or analyzed in this study.
